# Inflammatory biomarkers and cognitive functioning in individuals with euthymic bipolar disorder: exploratory study

**DOI:** 10.1192/bjo.2021.966

**Published:** 2021-07-09

**Authors:** Rebecca Strawbridge, Rowena Carter, Francesco Saldarini, Dimosthenis Tsapekos, Allan H. Young

**Affiliations:** Department of Psychological Medicine, Institute of Psychiatry, Psychology & Neuroscience, King's College London, UK; National Affective Disorders Service, South London & Maudsley NHS Foundation Trust, UK; Department of Psychological Medicine, Institute of Psychiatry, Psychology & Neuroscience, King's College London, UK; Department of Psychological Medicine, Institute of Psychiatry, Psychology & Neuroscience, King's College London, UK; Department of Psychological Medicine, Institute of Psychiatry, Psychology & Neuroscience, King's College London, UK; and National Affective Disorders Service, South London & Maudsley NHS Foundation Trust, UK

**Keywords:** Inflammation, cognition, bipolar affective disorder, neurogenesis, biomarker

## Abstract

**Background:**

Neurobiological research frequently implicates inflammatory and neurogenic components with core aspects of bipolar disorder. Even in periods of symptom remission (euthymia), individuals with bipolar disorder experience cognitive impairments, which are increasingly being proposed as an outcome for interventions; identifying biomarkers associated with cognitive impairment in people with bipolar disorder could advance progress in this therapeutic field through identifying biological treatment targets.

**Aims:**

We aimed to identify proteomic biomarker correlates of cognitive impairment in individuals with euthymic bipolar disorder.

**Method:**

Forty-four adults with a bipolar disorder diagnosis in euthymia underwent a battery of cognitive assessments and provided blood for biomarkers. We examined a comprehensive panel of inflammatory and trophic proteins as putative cross-sectional predictors of cognition, conceptualised according to recommended definitions of clinically significant cognitive impairment (binary construct) and global cognitive performance (continuous measure).

**Results:**

A total of 48% of the sample met the criteria for cognitive impairment. Adjusting for potentially important covariates, regression analyses identified lower levels of three proteins as significantly and independently associated with cognitive deficits, according to both binary and continuous definitions (interleukin-7, vascular endothelial growth factor C and placental growth factor), and one positively correlated with (continuous) global cognitive performance (basic fibroblast growth factor).

**Conclusions:**

This study identifies four candidate markers of cognitive impairment in bipolar disorder, none of which have been previously compared with cognitive function in participants with bipolar disorder. Pending replication in larger samples and support from longitudinal studies, these markers could have implications for treating cognitive dysfunction in this patient population.

## Cognitive impairment in BD

Bipolar disorder is a common, complex and costly illness with a poorly defined aetiology and, despite effective treatments,^1^ sufferers continue to experience long-term disability and reduced quality of life.^2^ The cause of disability in bipolar disorder is multifactorial and extends beyond the effect of acute manic or depressive episodes.^3^ A critical contributing factor to disability in bipolar disorder is cognitive impairment, which frequently persists even during periods of euthymic mood.^4,5^ These deleterious effects of cognitive dysfunction on quality of life are widespread, ranging from everyday psychosocial functioning (including occupational, household and social function),^5^ core illness outcomes (e.g. number and severity of affective episodes, including hospital admission rate)^5^ and suicidal ideation.^6^

The prevalence of clinically significant cognitive impairment in euthymic bipolar disorder is estimated to be between 30 and 57%, and is found across cognitive domains of memory, attention, processing speed and executive functioning;^7^ however, within these rates, there is significant heterogeneity as to the severity and domain specificity of impairments experienced.^4^ Although some current treatments may have some protective effects on cognition (e.g. lithium, lurasidone), intervention at present does not specifically target cognitive impairment in those with bipolar disorder, despite some emerging evidence of potential cognitive interventions.^8–10^ However, our understanding of the physiological mechanisms underlying cognitive impairment and bipolar disorder is lacking, which hinders progress in treatment options.

## Neurobiological mechanisms of cognitive impairment

Recent evidence suggests that cognitive difficulties in people with depression,^11^ schizophrenia,^12^ Parkinson's disease^13^ and Alzheimer's disease^14^ may be linked to inflammation. As with these other illnesses, bipolar disorder is understood to have an inflammatory component, with systematic reviews reporting elevated pro-inflammatory cytokines, such as tumour necrosis factor-*α* (TNF-*α*), soluble tumour necrosis factor receptor type 1 (sTNF-R1) and soluble inlerleukin-2 receptor (sIL-2R) levels in patients with manic bipolar disorder compared with controls; elevated sTNF-R1 levels in manic compared with euthymic bipolar disorder;^15^ and elevated C-reactive protein (CRP)^16^ and interleukin-6 (IL-6)^17^ levels in depression compared with euthymia. Despite this, altered immune responses have also been shown to persist even when patients are euthymic.^16,17^

The relationship between chronic inflammation and cognitive impairment is not fully understood, but several theories for other psychiatric illnesses have postulated that chronic inflammation results in hippocampal volume loss related to hypothalamic-pituitary-adrenal (HPA) axis dysregulation as a mechanism for poor verbal recognition memory in patients with depression.^18^ Microglial activation has been proposed as a potential mechanism of cognitive impairment in bipolar disorder,^19^ and immune-modulatory drugs have shown some promising initial results in improving cognition in schizophrenia.^20^ The relationship between inflammation and Alzheimer's disease has been more widely studied than associations in mood and psychotic disorders; in models of dementia, a neuroinflammatory state has been demonstrated to activate microglia and release cytokines,^21^ ultimately leading to neuronal loss and exacerbating A*β* and neurofibrillary pathologies.^22,23^ Neurogenesis is also likely to be intimately involved with these relationships, with growth factor markers being clearly associated with neurocognitive and inflammatory functions,^24^ and linked, alongside inflammation, with severity in mood disorders.^25^

## Biological links between affect and cognition

With increasing evidence that inflammation plays a critical role in the development of cognitive impairment across a range of psychiatric conditions, it is imperative this is considered more closely in bipolar disorder, given the burden of illness faced by patients with bipolar disorder who experience cognitive impairment. Evidence to date implicates some of the commonly assessed inflammatory markers in cognitive dysfunction, although this pertains to a limited set of biomarkers and the relationship with affective symptoms is unclear.^26–29^ Because of the difficulties disentangling specific cognitive impairment from impairments arising as a result of affective symptoms, it is important that cognitive impairment that would require treatment (i.e. be persistent beyond affective episodes) is assessed in fully euthymic states. Better understanding of the underlying pathophysiology may assist in developing/repurposing new treatment compounds or advancing the optimisation of existing treatment options in the future.

To our knowledge, no studies have yet compared a comprehensive panel of proteomic inflammatory markers and growth factor proteins with clinically relevant cognitive measures in bipolar disorder; this might facilitate a broader consideration of related biological networks as cognitive correlates in clinical practice. Besides, most studies of inflammation have only adjusted for limited clinical or demographic factors in these analyses, which limits the translational utility of findings.

## 

### Objectives

This study takes a comprehensive exploration of inflammatory predictors of cognitive impairment in adults with bipolar disorder not currently experiencing an episode of depression or (hypo)mania. We examine several demographic and clinical factors alongside a wide range of inflammatory and growth factor protein markers, defining cognitive performance according to international recommendations.^30^

Previous evidence has suggested that elevated pro-inflammatory cytokines and/or CRP partially explain severity of cognitive impairment, but several other constructs, not always adjusted for, are known to influence inflammation and be associated with bipolar disorder. As the majority of protein markers examined in this study have not previously been assessed in association with cognitive function in individuals with bipolar disorder, this is considered an exploratory study, the results of which can be used to guide future hypothesis-driven studies.

## Method

### Design

This study is a secondary analysis of cross-sectional (baseline) data from the Cognitive Remediation in Bipolar (CRiB) study.^8^ Methodological details of the CRiB study have been described previously.^8,31^ The CRiB study investigated a sample of 60 participants; the present analysis focuses on a subsample of 44 individuals who provided blood for biomarker analysis (*N* = 44), which was an optional assessment in the CRiB study.

### Participants

Individuals were included in the study if they had a diagnosis of bipolar disorder (type 1 or type 2), had been in a euthymic affective state for at least 1 month, were aged 18–65 years, fluent in English and did not have a current substance use or personality disorder, or an impairing organic neurological disorder. Participants already had a formal bipolar disorder diagnosis, which was validated with the MINI-International Neuropsychiatric Interview (MINI).^32^ The MINI was also used to ensure an absence of substance use disorders. To qualify as euthymic, participants needed to meet the Newcastle Euthymia Protocol criteria, scoring ≤7 on the Hamilton Rating Scale for Depression (HRSD)^33^ and Young Mania Rating Scale (YMRS)^34^ at two time points 1 week apart, covering the month before inclusion.

### Procedure

The study had received prior approval from the UK's Health Research Authority and London City Road & Hampstead NHS Research Ethics Committee (identifier 15/LO/1557; trial registration ISRCTN-32290525). Participants were recruited via community advertisement and primary and secondary care healthcare services, and all provided written informed consent before taking part. The data examined in this study was then provided in a single session, before participants were randomised to receive a cognitive remediation intervention or continue treatment as usual.

### Measures

#### Clinical, psychosocial and demographic

Continuous variables assessed were age, body mass index, number of medications currently taken, health-related quality of life (as measured by the EuroQol-5D questionnaire^35^), number of lifetime affective episodes, psychosocial functioning (measured by the Functioning Assessment Short Test (FAST)^36^), subsyndromal symptoms of depression (measured by the HRSD^33^) and mania (measured by the YMRS^34^), anxiety symptoms (measured by the Hamilton Rating Scale for Anxiety (HRSA)^37^) and history of childhood trauma (measured by the Childhood Trauma Questionnaire (CTQ)^38^). Binary variables assessed were gender (all participants identifying as male or female based on free-text self-report), type of bipolar disorder (type 1 or type 2), current physical illness (yes/no), alcohol use (nil/low or medium/high, as per thresholds on the MINI interview^32^) and smoking (yes/no). These ‘non-biological’ factors were selected *a priori* according to their understood associations with cognition and/or inflammation, as well as availability from the primary study, and all were considered in analyses as described in the statistical analysis section below.

#### Cognitive

The CRiB study included measurement of a neuropsychological battery producing numerous cognitive variables, as described previously.^8,31^ To reduce the (already extensive) number of comparisons undertaken, two measures of cognition were computed as informed by the International Society of Bipolar Disorders Cognitive Taskforce recommendations.^30^ These essentially measure global cognitive performance as a continuous measure, and cognitive impairment as a classified (dichotomous) construct.^39^ The continuous measure of ‘global’ cognitive performance (higher scores indicating less impairment) is calculated from eight cognitive tests across four domains: processing speed (using the Digit Symbol Substitution Test and symbol search (Wechsler Adult Intelligence Scale)^40^), working memory (using the digit span (Wechsler Adult Intelligence Scale)^40^), verbal learning and memory (from the verbal paired associates tests I and II (Wechsler Memory Scale)^41^), and executive functioning (from the Hotel test,^42^ matrix reasoning (Wechsler Abbreviated Scale of Intelligence)^43^ and verbal fluency F-A-S test^44^). For each test, the raw score was transformed into standardised normative scores (correcting for age and education) as per test manuals, and the composite global score was then calculated by averaging each participant's *z*-scores across individual cognitive tests.^4^ The binary summary variable representing clinically significant cognitive impairment^30^ categorises participants scoring ≥1 s.d. below published norms on two or more of the aforementioned cognitive tests as impaired and others as unimpaired.

#### Biomarker

For each participant, 5 mL of blood was drawn. Plasma was obtained after centrifugation and samples were stored at −80 until thawing for assay. Plasma samples were assayed using a high-sensitivity Meso Scale Discovery (MSD) V-Plex kit (Meso Scale Diagnostics, Maryland, USA) as previously.^45^ This kit was selected because of its ability to assess an extensive range of inflammatory and trophic proteins understood to be relevant to affective disorders and with a high detection sensitivity.^25,45^ The panel of protein markers comprised brain-derived neurotrophic factor (BDNF), basic fibroblast growth factor (bFGF), CRP, eotaxin, eotaxin-3, fms-like tyrosine kinase (Flt-1), intracellular adhesion module (ICAM-1), interferon-*γ* (IFN-*γ*), interleukin-10 (IL-10), interleukin-12 (IL-12), interleukin-15 (IL-15), interleukin-16 (IL-16), interleukin-17 (IL-17), interleukin-1*α* (IL-1*α*), IL-6, interleukin-7 (IL-7), interleukin-8 (IL-8), interferon-*γ-*induced protein-10 (IP-10), macrophage chemoattractant protein-1 (MCP-1), macrophage chemoattractant protein-4 (MCP-4), macrophage inflammatory protein-1*α* (Mip-1*α*), macrophage inflammatory protein-1*β* (Mip-1*β*), placental growth factor (PlGF), serum amyloid-A (SAA), thymus and activation-regulated chemokine (TARC), angiopoietin-1 receptor (Tie-2), TNF-*α*, tumour necrosis factor-*β* (TNF-*β*), vascular cell adhesion molecule-1 (VCAM-1), vascular endothelial growth factor (VEGF), vascular endothelial growth factor C (VEGF-C) and vascular endothelial growth factor D (VEGF-D). Plasma was assayed with the MSD array, according to manufacturer's manual, with seven-point standard curves run in duplicate to calculate the levels of each protein with each sample, and a no-template control included to control for background fluorescence. The standard curves demonstrated very high concentration-fluorescence correlations (*r*^2^ > 0.99), indicating reliability. Protein levels are expressed as picograms per millilitre, unless otherwise stated.

### Statistical analyses

Protein levels were transformed (log base 10) and normality of distribution assessed (via skewness and kurtosis values, visual inspection of stem and leaf diagrams and box plots in addition to the Kolmogorov–Smirnov test). Because of issues surrounding the removal of outliers, particularly in small samples, bootstrapping of 1000 samples was employed on all statistical tests instead, because in these cases outliers hold less weight without removing potentially valid data altogether, and this also has the benefit of dealing with slightly non-normally distributed variables.^46^ Initially, univariate indications of proteins associated with the two cognitive variables was assessed with Spearman's correlation (for global cognitive performance) and *t*-tests (for cognitive impairment*).* Any protein associated with either cognitive variable at *P* < 0.1 (‘potentially indicative’) was decided *a priori* to be considered further as putative predictors of cognitive function in respective multivariable models. Before multivariable models, the respective univariate tests also compared the above-mentioned ‘non-biological factors’ selected as putative confounders with the cognitive outcomes, and with the indicated protein markers; any associated at *P* < 0.01 were also included as covariates in these multiple regression analyses. Multiple regressions thus aimed to explore all potentially indicative markers (i.e. biological and non-biological factors associated with cognition at *P* < 0.1 in univariate tests, as independent variables) of each cognitive outcome (dependent variable). Both linear regressions (predicting global cognitive performance) and logistic regressions (predicting impairment status) were conducted. Note that the terms ‘predicting’ or ‘predictors’ here refer to cross-sectional statistical associations, rather than longitudinal prediction. Model assumptions for collinearity were checked (Hosmer–Lemeshow test in logistic regressions, and Durbin–Watson in linear regressions) and a *P*-value of <0.05 was considered nominally significant.

## Results

### Sample characteristics

Forty-four participants were assessed (based on protein marker availability in addition to the study eligibility criteria). Participants who were not currently well on the day of the baseline assessment rescheduled their appointment, and therefore, to our knowledge, none had a cold or current infection. Twenty-one participants were grouped as cognitively impaired (48%) and 23 participants were grouped as unimpaired. The mean composite cognitive performance scores were *z* = −0.675 (s.d. 0.426) for the impaired group and *z* = 0.261 (s.d. 0.350) for the unimpaired group (overall mean *z* = −0.186, s.d. = 0.609 across the sample). Supplementary Tables 1 and 2 available at https://doi.org/10.1192/bjo.2021.966 contain descriptive data related to inflammation and cognition respectively. Clinical and demographic characteristics of the sample are presented in Table 1. None of the non-biological variables had any missing values. Four proteins (IFN-*γ*, IL-10, TARC and TNF-*β*) had 1–19% levels undetected by the assay, and these missing values were imputed with half the lower limit of detection.^47^ Eight proteins had ≥20% levels undetected by the assay (granulocyte-macrophage colony-stimulating factor, interleukin -2p70, -13, -1*β*, -2, -4 and -5, and interferon-*α*) and were excluded from analyses (see Supplementary Table 1); these rates of non-detection align with previous reports in similar samples.^25,45^ A total of 32 proteins were analysed.

**Table 1:** Participant characteristics

Characteristics		All, *N* = 44	Cognitive groups	
			Impaired, *n* = 21	Unimpaired, *n* = 23
Continuous measures, mean (s.d.)				
Age		43.7 (12.8)	44.3 (13.0)	43.9 (13.2)
Number of medications currently taken		3.7 (2.2)	3.4 (2.0)	3.0 (2.4)
BMI^a^		27.8 (6.1)	28.8 (5.7)	26.8 (6.3)
Health-related quality of life (EuroQol-5D score)		6.9 (1.7)	6.9 (1.8)	6.9 (1.6)
Number of episodes		20.7 (17.2)	22.4 (20.1)	20.3 (14.8)
Depressive symptoms (HRSD score)		4.0 (2.7)	4.0 (2.5)	4.0 (3.0)
Manic symptoms (YMRS score)		2.7 (2.40)	3.0 (2.2)	2.6 (2.6)
Level of functioning (FAST score)		20.8 (9.6)	24.0 (9.4)	17.9 (9.1)
Anxiety symptoms (HRSA score)		6.4 (4.4)	5.9 (3.1)	6.6 (5.5)
Childhood adverse experience (CTQ score)		44.0 (16.8)	44.8 (20.6)	44.2 (13.3)
Binary measures, *n* (%)				
Gender	Male	13 (29.5%)	5 (24%)	8 (35%)
	Female	31 (70.5%)	16 (76%)	15 (65%)
Bipolar disorder type	Type 1	26 (59%)	13 (62%)	13 (57%)
	Type 2	18 (41%)	8 (38%)	10 (43%)
Physical illness	No	18 (41%)	7 (33%)	11 (48%)
	Yes	26 (59%)	14 (67%)	12 (52%)
Smoking^b^^,^^c^	No	32 (73%)	12 (57%)	20 (87%)
	Yes	12 (27%)	9 (43%)	3 (13%)
Alcohol use^d^	No/low	26 (59%)	14 (67%)	12 (52%)
	Mid/high	18 (41%)	7 (33%)	11 (48%)

BMI, body mass index; HRSD, Hamilton Rating Scale for Depression; YMRS, Young Mania Rating Scale; FAST, Functioning Assessment Short Test; HRSA, Hamilton Rating Scale for Anxiety; CTQ, Childhood Trauma Questionnaire.

a. Data missing for two participants. No other missing data.

b. Originally smoking coded as current/past/never, but previous smokers did not differ from those who had never smoked in any cognitive or inflammatory measure and the two were pooled.

c. The only significant association with cognition was that participants with cognitive impairment were more frequently smokers (*χ*^2^ = 4.91, *P* = 0.027).

d. Alcohol use coded according to the MINI interview. Cognitive impaired/unimpaired groups were compared with participant characteristics, using *t*-tests (continuous) and chi-squared tests (binary). Composite cognitive performance were compared with participant characteristics, using Spearman's correlation (continuous) and t-test (binary).

### Univariate associations between proteins and cognition

**Fig. 1:**
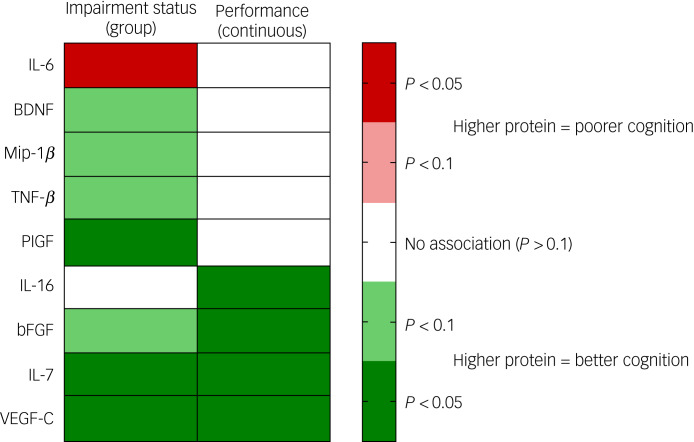
Summary of univariate associations between cognitive and protein markers. BDNF, brain-derived neurotrophic factor; bFGF, basic fibroblast growth factor; IL-6, interleukin-6; IL-7, interleukin-7; IL-16, interleukin-16; Mip-1*β*, macrophage inflammatory protein-1*β*; PlGF, placental growth factor; TNF-*β*, tumour necrosis factor-*β*; VEGF-C, vascular endothelial growth factor C.

Table 2 displays the univariate associations between biomarker and cognitive variables. Twenty-three proteins were not associated at *P* < 0.1 with cognitive summary measures; nine proteins were indicated as potentially associated with at least one of the cognitive variables, summarised below and in Fig. 1.

**Table 2:** Univariate associations between proteins and cognitive function

Biomarker	Impaired/non-impaired group comparisons			Global cognitive performance comparisons	
	*t*-value	*P*-value	95% CI	*r*-value	*P*-value
Growth factors					
BDNF	−1.948	0.058	−0.311 to 0.005*	0.137	0.377
bFGF	−1.713	0.094	−0.471 to 0.038*	0.333	0.027**
Flt-1	−0.490	0.627	−0.062 to 0.038	0.182	0.238
PlGF	−2.016	0.050	−0.147 to 0.000**	0.212	0.166
Tie-2	−0.934	0.356	−0.116 to 0.043	0.070	0.653
VEGF-C	−2.152	0.037	−0.269 to −0.009**	0.333	0.027**
VEGF-D	−1.373	0.177	−0.130 to 0.025	0.245	0.110
VEGF	−1.377	0.176	−0.191 to 0.036	0.171	0.267
Inflammatory markers					
CRP	0.393	0.697	−0.270 to 0.401	−0.118	0.445
Eotaxin	−0.798	0.429	−0.122 to 0.053	−0.048	0.759
Eotaxin-3	−0.840	0.406	−0.132 to 0.054	−0.012	0.940
ICAM-1	0.262	0.794	−0.078 to 0.101	0.041	0.789
IFN-*γ*	0.231	0.818	−0.275 to 0.347	0.124	0.420
IL-10	−0.898	0.374	−0.367 to 0.141	0.074	0.633
IL-12	−1.084	0.285	−0.205 to 0.062	0.077	0.821
IL-15	−0.973	0.336	−0.058 to 0.020	0.162	0.294
IL-16	−1.556	0.127	−0.146 to 0.019	0.297	0.050**
IL-17	−0.436	0.665	−0.149 to 0.096	0.032	0.839
IL-1*α*	1.000	0.323	−0.114 to 0.339	0.228	0.136
IL-6	1.877	0.067	−0.015 to 0.422*	−0.140	0.366
IL-7	−3.828	<0.001	−0.475 to −0.147****	0.395	0.008***
IL-8	−0.656	0.516	−0.206 to 0.105	0.210	0.172
IP-10	−0.099	0.922	−0.147 to 0.134	−0.049	0.752
MCP-1	−0.291	0.773	−0.099 to 0.074	−0.098	0.527
MCP-4	−1.006	0.320	−0.100 to 0.033	0.006	0.968
Mip-1*α*	−0.433	0.667	−0.122 to 0.079	−0.001	0.996
Mip-1*β*	−1.792	0.080	−0.196 to 0.012*	0.097	0.533
SAA	−1.441	0.157	−0.456 to 0.076	0.138	0.372
TARC	−0.960	0.343	−0.896 to 0.318	0.247	0.106
TNF-*α*	−0.331	0.742	−0.125 to 0.089	0.076	0.626
TNF-*β*	−1.841	0.073	−0.436 to 0.020*	0.112	0.468
VCAM-1	−0.961	0.342	−0.135 to 0.048	0.084	0.590

Positive *t*-values for the impairment group indicates higher protein levels in participants with cognitive impairment (negative *t*-values indicate lower protein levels in participants with cognitive impairment). A positive correlation between protein and cognitive performance indicates higher protein levels in those with better cognitive performance. BDNF, brain-derived neurotrophic factor; bFGF, basic fibroblast growth factor; Flt-1, fms-like tyrosine kinase; PlGF, placental growth factor; Tie-2, angiopoietin-1 receptor; VEGF-C, vascular endothelial growth factor C; VEGF-D, vascular endothelial growth factor D; VEGF, vascular endothelial growth factor; CRP, C-reactive protein; ICAM-1, intracellular adhesion module; IFN-*γ*, interferon-*γ*; IL-10, interleukin-10; IL-12, interleukin-12; IL-15, interleukin-15; IL-16, interleukin-16; IL-17, interleukin-17; IL-1*α*, interleukin-1*α*; IL-6, interleukin-6; IL-7, interleukin-7; IL-8, interleukin-8; IP-10, interferon-*γ-*induced protein-10; MCP-1, macrophage chemoattractant protein-1; MCP-4, macrophage chemoattractant protein-4; Mip-1*α*, macrophage inflammatory protein-1*α*; Mip-1*β*, macrophage inflammatory protein-1*β*; SAA, serum amyloid-A; TARC, thymus and activation-regulated chemokine; TNF-*α*, tumour necrosis factor-*α*; TNF-*β*, tumour necrosis factor-*β*; VCAM-1, vascular cell adhesion molecule-1.

*P < 0.1, **P < 0.05, ***P < 0.01, ****P < 0.001.

VEGF-C and IL-7 were significantly associated with both measures of cognition, with higher levels in the cognitively unimpaired group and a positive correlation with cognitive performance. The same pattern was observed for bFGF, although the association with the group was non-significant at *P* < 0.05. IL-16 was also positively correlated with continuous performance, but not to a significant extent with impairment group. PlGF was also higher in the cognitively unimpaired group, but not correlated to a significant extent with continuous performance. Three further markers were non-significantly (*P* < 0.1) higher in participants without cognitive impairment (BDNF, TNF-*β*, Mip-1*β*). IL-6 was the only marker found to be higher in participants with cognitive impairment, although the association did not meet the threshold for statistical significance.

Because of the number of comparisons, the above analyses are to be interpreted as only preliminary, non-inferential indications. Before regression analyses, the nine indicated proteins were compared with non-biological variables, presented in Supplementary Tables 2 and 3. The only non-biological markers associated with cognition were the FAST measure of psychosocial functioning, which was tentatively and positively associated with global cognitive performance (*r* = −0.275, *P* = 0.071), but not impairment group; and smoking, which was more prevalent in participants grouped as cognitively impaired versus unimpaired (*χ*^2^ = 4.91, *P* = 0.027), but was not associated with global performance as a continuous measure. Multivariable models included FAST in all linear regressions, and smoking in all logistic regressions.

### Multivariable regression analyses

Tables 3 and 4 present the results of regression models predicting, cross-sectionally, cognitive group status (logistic; Table 3) and the continuous measure of global cognitive performance (linear; Table 4) with each of the nine indicated proteins as independent variables.

**Table 3:** Multivariable logistic regressions predicting cognitive impairment group

Biomarker	Model *r*^2^	Model *χ*^2^	Model *P*	Independent variables	Odds ratio	95% CI	*P-*value
BDNF	0.307	11.519	**0.009**	BDNF	0.166	0.010–2.782	0.176
				Number of medications	1.324	0.933–1.878	0.100
				**Smoking**	7.381	1.322–41.21	**0.004**
bFGF	0.225	8.135	0.017	bFGF	0.252	0.050–1.281	0.110
				**Smoking**	5.311	1.146–24.61	**0.015**
IL-16^a^	0.234	8.467	0.132	IL-16	0.010	0.000–3.138	0.239
				HRQOL	0.942	0.586–1.515	0.845
				CTQ	1.007	0.962–1.054	0.754
				Bipolar type	1.626	0.392–6.748	0.539
				**Smoking**	5.336	1.101–25.87	**0.018**
IL-6^a^	0.383	14.895	**0.011**	IL-6	3.522	0.267–46.45	0.320
				**HRQOL**	0.453	0.233–0.883	**0.001**
				Smoking	0.166	0.024–1.166	0.060
				Physical illness	0.270	0.047–1.537	0.099
				**FAST**	1.111	0.997–1.239	**0.020**
IL-7	0.465	18.878	**<0.001**	**IL-7**	0.018	0.001–0.369	**0.005**
				Number of medications	1.290	0.889–1.871	0.244
				**Smoking**	7.727	1.142–52.27	**0.007**
Mip-1*β*	0.202	7.216	0.065	Mip-1*β*	0.049	0.001–3.910	0.170
				Gender	1.162	0.243–5.569	0.840
				**Smoking**	4.385	0.946–20.33	**0.027**
PlGF^a^	0.471	19.162	**0.004**	**PlGF**	0.000	0.000–0.265	**0.023**
				**Number of medications**	1.748	1.087–2.810	**0.013**
				HRQOL	0.764	0.454–1.287	0.371
				Gender	1.648	0.247–10.98	0.605
				**Smoking**	12.26	1.408–106.7	**0.003**
				Age	0.984	0.984–1.180	0.100
TNF-*β*	0.246	8.958	**0.030**	**TNF-*β***	0.171	0.025–1.194	**0.030**
				Number of episodes	0.991	0.953–1.030	0.668
				**Smoking**	5.765	1.198–27.73	**0.023**
VEGF-C	0.297	11.084	**0.004**	**VEGF-C**	0.022	0.001–0.590	**0.006**
				**Smoking**	7.730	1.329–44.94	**0.011**

Multivariable logistic regressions did not indicate a significant concern of collinearity within any of the models (Hosmer–Lemeshow test). The *P*-values provided are following bootstrapping. Bold text indicates significance at *P* < 0.05. BDNF, brain-derived neurotrophic factor; bFGF, basic fibroblast growth factor; IL-16, interleukin-16; HRQOL, health-related quality of life (EQ-5D score); CTQ, childhood trauma severity (Childhood Trauma Questionnaire); IL-6, interleukin-6; FAST, functional impairment (Functioning Assessment Short Test); IL-7, interleukin-7; Mip-1*β*, macrophage inflammatory protein-1*β*; PlGF, placental growth factor; TNF-*β*, tumour necrosis factor-*β*; VEGF-C, vascular endothelial growth factor C.

a. For underpowered models (those containing more than one independent variable per ten participants, i.e. more than four in total), regressions were re-run containing only covariates that were significant at *P* < 0.05 (between inflammatory and non-biological, or cognitive and non-biological). The results were similar; IL-6 differed in that health-related quality of life was no longer significantly associated with impairment group.

**Table 4:** Multivariable linear regressions predicting global cognitive performance

Biomarker	Model adjusted *r*^2^	Model *F*	Model *P*	Independent variables	Standardised *β*-value	95% CI	*P-*value
BDNF	0.039	1.580	0.209	BDNF	0.096	−0.517 to 0.954	0.493
				Number of medications	−0.120	−0.124 to 0.058	0.454
				FAST	−0.213	−0.034 to 0.007	0.121
bFGF	0.158	5.035	**0**.**011**	**bFGF**	0.344	0.085–0.894	**0**.**007**
				**FAST**	−0.253	−0.034 to 0.002	**0**.**037**
IL-16^a^	0.079	1.741	0.149	IL-16	0.249	−0.390 to 2.593	0.194
				HRQOL	0.185	−0.085 to 0.221	0.528
				CTQ	−0.082	−0.016 to 0.010	0.676
				Bipolar type	−0.018	−0.412 to 0.367	0.907
				**FAST**	−0.380	−0.047 to −0.002	**0**.**037**
IL-6	0.060	1.552	0.197	IL-6	−0.095	−0.762 to 0.447	0.607
				HRQOL	0.388	−0.005 to 0.290	0.103
				Smoking	−0.063	−0.519 to 0.349	0.739
				Physical illness	−0.174	−0.627 to 0.202	0.238
				**FAST**	−0.412	−0.049 to −0.003	**0**.**025**
IL-7	0.176	4.067	**0**.**013**	**IL-7**	0.381	0.181–1.322	**0**.**003**
				Number of medications	−0.060	−0.100 to 0.067	0.661
				FAST	−0.203	−0.032 to 0.006	0.117
Mip-1*β*	0.035	1.524	0.223	Mip-1*β*	0.127	−0.789 to 1.672	0.459
				Gender	−0.047	−0.513 to 0.388	0.719
				**FAST**	−0.315	−0.040 to 0.000	**0**.**028**
PlGF	0.180	2.348	**0**.**044**	**PlGF**	0.426	0.298–3.861	**0**.**048**
				Number of medications	−0.183	−0.138 to 0.037	0.260
				HRQOL	0.301	−0.019 to 0.240	0.116
				Gender	−0.017	−0.432 to 0.386	0.919
				Smoking	−0.730	−0.607 to 0.286	0.498
				Age	−0.063	−0.035 to 0.001	0.113
				**FAST**	−0.354	−0.044 to −0.001	**0**.**039**
TNF-*β*	0.050	1.758	0.171	TNF-*β*	0.195	−0.177 to 0.794	0.217
				Number of episodes	0.036	−0.010 to 0.012	0.831
				**FAST**	−0.312	−0.040 to 0.000	**0**.**031**
VEGF-C	0.185	5.866	**0**.**006**	**VEGF-C**	0.381	0.275–1.815	**0**.**006**
				**FAST**	−0.338	−0.039 to −0.004	**0**.**011**

Multivariable linear regressions did not indicate a significant concern of collinearity within any of the models (Durbin–Watson value between 1 and 3.) Bold text indicates significance at *P* < 0.05. BDNF, brain-derived neurotrophic factor; FAST, functional impairment (Functioning Assessment Short Test); bFGF, basic fibroblast growth factor; IL-16, interleukin-16; HRQOL, health-related quality of life (EQ-5D score); CTQ, childhood trauma severity (Childhood Trauma Questionnaire); IL-6, interleukin-6; IL-7, interleukin-7; Mip-1*β*, macrophage inflammatory protein-1*β*; PlGF, placental growth factor; TNF-*β*, tumour necrosis factor-*β*; VEGF-C, vascular endothelial growth factor C.

a. For underpowered models (those containing more than one independent variable per ten participants, i.e. more than four in total), regressions were re-run only containing covariates that were significant at *P* < 0.05 (between inflammatory and non-biological, or cognitive and non-biological). The results were similar, with the exception of PlGF, for which the model as a whole was no longer significant.

As in the univariate associations, both VEGF-C and IL-7 were significantly lower in participants with poorer cognition in both cognitive outcomes (*P* < 0.01). In both logistic regressions, smoking also remained a significant predictor of impairment. IL-7 was the only significant independent predictor in the relevant linear regression, whereas VEGF-C was accompanied by FAST, which was also significant at *P* < 0.05. bFGF was significantly lower (*P* < 0.01) in those with poorer cognitive performance (with FAST also significant at *P* < 0.05), but the association with impairment group in logistic regression was not significant. IL-16 did not predict better cognitive performance or unimpaired group status when considered in regressions alongside health-related quality of life, childhood trauma severity, bipolar type and FAST/smoking.

Of the four proteins (PlGF, BDNF, Mip-1*β* and TNF-*β*) that indicated a univariate association with group (higher in participants without cognitive impairment) but not continuous performance, only PlGF contributed significantly (*P* < 0.05) to both cognitive outcomes in multivariable regression models (also containing medications, health-related quality of life, gender, smoking, age and FAST). The other three biomarkers were not significantly associated with cognitive performance. BDNF and Mip-1*β* also did not predict impairment group status, but TNF-*β* (alongside number of episodes and smoking) significantly predicted impairment status at *P* < 0.05.

IL-6 was the only protein indicated at *P* < 0.1 as higher in the impaired versus unimpaired group. In multivariable regressions (adjusting for health-related quality of life, smoking, physical illness and FAST), this cytokine was not a significant predictor of either cognitive outcome, although was the only protein indicating an effect of medium (as opposed to small) effect size (odds ratio 3.52).

### *Post hoc* analyses

We maximised inclusiveness of eligibility criteria for this secondary analysis because of the small sample size. This meant that, *a priori,* participants with autoimmune illnesses or taking anti-inflammatory medications were not excluded. However, since these clearly affect inflammatory marker levels (and also may influence cognition), these factors were considered *post hoc*. After data were accessed, two participants were identified who had an autoimmune condition (*n* = 1) or were taking anti-inflammatory medications (*n* = 2). Sensitivity analyses were conducted, reanalysing the above comparisons with these two participants removed. Results were largely unaffected by this (differences are described in Supplementary Table 4), with the main changes being that TNF-*β* and IL-6 no longer indicated association with cognitive functioning. In the multivariable models, the same cytokines were significant as in the planned analyses, with one exception (TNF-*β*; significance reduced to *P* = 0.057).

An exploratory comparison employed Spearman's correlation to indicate associations between this subset of putative biomarkers with the individual cognitive domains that comprised the cognitive summary variables (see Supplementary Table 4). Most correlations between proteins and individual domains were small and the only associations where *P* < 0.01 were IL-7 with verbal memory (Verbal Paired Associates II) and processing speed (symbol search), and VEGF-C with symbol search. Four biomarkers (IL-6, Mip-1*β*, PlGF and TNF-*β*) were not significantly associated with any individual domain, and executive functioning was not significantly associated with any protein levels.

## Discussion

In this sample of 44 euthymic participants with bipolar disorder, 6 of 32 examined proteins were associated with composite cognitive outcomes and 4 remained significant in regression models, after adjusting for potentially relevant non-biological covariates (both when including and excluding participants with an inflammatory condition or anti-inflammatory medication).

Three of these (VEGF-C, IL-7 and PlGF) were all lower in participants with poorer cognition as measured by both outcomes examined (clinically relevant impairment and global cognitive performance). With the same direction of effect, bFGF was predictive of global performance and, to a lesser extent, TNF-*β* was predictive of impairment group status cross-sectionally.

### Neurobiology, mood and cognition in bipolar disorder

It might be expected that neurobiological dysregulations in bipolar disorder would be confined to mood episodes, and most research has investigated pro-inflammatory states during mania or depression.^48,49^ However, elevated inflammatory^50^ and attenuated trophic^51^ biomarkers have also been reported in periods of euthymia. Within-participants comparisons of these biomarkers across different affective states are scarce, but suggest more pronounced dysregulations in mood states.^49,52,53^ Thus, we may have identified stronger or more frequent biomarker associations if the study been conducted when participants were experiencing an episode. However, our main interest was in cognitive associations with these biomarkers, and focusing on individuals with euthymia allowed an investigation essentially independent of mood, which has been subject to more intensive research. It is notable that none of the nine proteins assessed were correlated with subsyndromal manic, depressive or anxious symptom severity.

To date, relatively few studies have explored the relationship between inflammation and cognition, as recently reviewed.^26^ A 2018 study reported that in the presence of poor performance in tasks of affective processing, verbal memory, working verbal memory and executive functioning, participants with euthymic bipolar disorder had elevated plasma pro- and anti-inflammatory cytokines compared with controls, with IL-6 being negatively correlated with global cognitive performance.^27^ A subsequent study reported that another prominent pro-inflammatory cytokine, TNF-*α* (but not IL-6 or other cytokines), was elevated in patients with poorer global cognition, processing speed and working memory.^28^ This association has recently also been reported for CRP,^29^ although the patients in the latter two studies were in variable affective states at the time of assessments, and the biomarker/cognitive assessments were not always collected on the same day. Previous studies tended to assess cognitive function by using domain-specific outcomes, often testing several individual cognitive tests, rather than defining cognitive impairment by using consensus-recommended definitions or computing global cognition composite scores.^30^ Critically, they also examined a limited set of inflammatory biomarkers. In measuring a comprehensive panel of proteins, we captured a broader neurobiological impression of inflammatory and growth factor markers whose functions are understood to interact.

### Candidate biomarkers of cognitive dysfunction in bipolar disorder

Our results only tentatively support an association, as identified previously,^27^ between elevated IL-6 and impaired cognition. Mechanistic evidence suggests that not only can this cytokine cross the blood–brain barrier, but it may be directly involved in memory consolidation.^54^ However, as supported by our analyses, it may be that this pro-inflammatory cytokine is more directly associated with well-being (e.g. quality of life or psychosocial functioning, which are putatively associated with reduced cognition) or affective symptoms. IL-6 has been found to rise particularly in mania,^49^ and may even fluctuate in association with subclinical symptoms in samples categorised as broadly euthymic.

BDNF is well-understood to be critical in cognitive function via neural plasticity and affected by inflammatory states,^19^ so it is unsurprising that this neurotrophic factor has been found as attenuated in the presence of cognitive impairment in bipolar disorder.^55^ Known to be particularly implicated in memory synthesis via neuronal action in the hippocampus, cortex and basal forebrain,^56^ it is possible that the BDNF–cognition association in our study did not reach significance because cognition was measured as a global construct, rather than domain-specific comparisons. However, this growth factor has often been strongly correlated across a variety of cognitive domains.^55,57^

bFGF, another neurotrophic factor and signalling protein involved in tissue repair and angiogenesis, has been found to enhance hippocampal neurogenesis following brain injuries.^58^ The positive association between bFGF and cognitive performance was significant only for the continuous measure and not for impairment group. To our knowledge, this is the first study assessing bFGF and cognition in bipolar disorder.

VEGF growth factors are signalling proteins involved in the growth and maintenance of both vascular and neural cells, and appear protective against cognitive impairment,^59^ particularly in the context of Alzheimer's disease.^60^ Notably, VEGF-A and VEGF-D were not associated with cognitive functioning in this study, but VEGF-C was markedly higher in participants with unimpaired cognitive performance (according to both cognitive outcomes). To our knowledge, VEGF-C has not been compared with cognitive function in individuals with bipolar disorder, but our results are supported by recent preclinical evidence of decreased cerebrospinal VEGF-C having a negative effect on cognitive task performance.^61^

PlGF is a ligand of VEGFR and is involved in the recruitment of monocytes and macrophages, which promote vessel growth and angiogenesis.^25^ Similar to VEGF-C above, we identified a significant and positive association between PlGF and overall cognitive performance, although this was not apparent when assessing individual cognitive domains, and we are not aware of previous studies of PlGF in people with bipolar disorder. Unlike PlGF, individual cognitive domain examinations supported a positive relationship across multiple tests of processing speed and memory with IL-7 and VEGF-C.

Similar to the above, IL-7 (which acts as a growth factor and cytokine important for B and T cell development) was positively and strongly significantly associated with cognitive function in both outcomes, in this study. Our findings accord with its understood mechanisms of action, although it has not, to our knowledge, previously been studied in association with cognition in bipolar disorder.

We are also not aware of previous comparisons between lymphotoxin (or TNF-*β*) and cognition in a sample of participants with euthymic bipolar disorder. TNF-*β* levels were lower in participants with cognitive impairment, which is slightly surprising given its functional proximity to TNF-*α* and sTNF receptors. which have documented involvement in both bipolar disorder and cognitive impairments, with effects in the reverse direction.^15,26,28^ Despite this, TNF-*β* has previously been reported as attenuated in the presence of inflammatory signals in those with severe depressive episodes,^62^ and we highlight that the association we identified with this marker was not robust (i.e. did not persist to a significant extent after exclusion of participants whose inflammatory activity was likely influenced by a health condition or medication).

The chemokine Mip-1*β* (CCL4) is produced in response to pro-inflammatory cytokines, and elevated levels have been reported in people with bipolar disorder with lower cortical thickness,^63^ as well as severity of cognitive impairment after stroke.^64^ In this study, Mip-1*β* was non-significantly lower in participants with cognitive impairment (and was also positively correlated with PlGF), which warrants further examination. It is worth noting that attenuated levels of this chemokine have been reported in people with depression (who typically present with pro-inflammatory indications) compared with healthy controls.^65^

When examining associations between these markers and individual cognitive domains *post hoc*, memory and processing speed were frequently correlated with these proteins, but a striking absence of significant relationships with executive functioning is noted, contrary to previous assertions.^26^

### Methodological considerations

As below, we first emphasise that this exploratory study was underpowered and that numerous statistical comparisons were undertaken without adjusting for multiple testing. Therefore, clearly all nominally significant findings from the current investigation require replication in larger samples of individuals with euthymic bipolar disorder with and without cognitive impairment, and including a matched group of non-affected controls. Our study was considered an exploratory investigation of a large panel of inflammatory and trophic proteins, many of which had not been subject to examination in samples with bipolar disorder, in only 44 participants. Thus, the comparisons made were underpowered statistically, and it is possible that type 2 errors may help to explain non-significant associations in this study that contrast with established markers of impaired cognition (e.g. BDNF). Proteins identified in this study as candidate biomarkers of cognitive dysfunction need to be considered in a unified predictive model controlling for more non-biological factors, to assess the predictive value of each putative marker as well as account for their inter-associations. Our analysis tentatively suggests that modelling a binary construct (i.e. clinically significant cognitive impairment) rather than a continuous global cognitive outcome might facilitate the identification of cognitive biomarkers and subsequent neurobiological treatment targets for improving cognition in this population.

One of the methodological issues common to neurobiological and neuropsychological research is the number of data points (or markers) required to fully ascertain the complexities of constructs such as ‘inflammation’ or ‘cognitive function’. Batteries of cognitive assessment commonly contain measurements of short-term and working memory, attention and executive functions, but there is much variation is the number and focus of tasks covering the umbrella of executive function domains. Typically, a well-rounded cognitive battery might be expected to comprise five to ten outcome variables. However, inflammatory research tends to focus on a few (two to five) traditional pro-inflammatory/T1 proteins.

Despite this, there are several other relevant constructs that we were not able to assess in this study, including waist circumference (which may better reflect adipose tissue, closely related to inflammation better than body mass index), biological gender in addition to or instead of identified gender, and use of specific medications that may have particular influence on cytokines (as opposed to general medication load). Another factor to consider is the imputation of half the limit of assay detection for proteins not detected by the MSD kit; although it is usual practice to make the assumption that this represents a low protein level present in the blood, it is indeed possible that there may have been other reasons for non-detection, and we note here that some putative biomarkers could not be assessed because of a particularly high rate of non-detection (including interleukin-1 (IL-1), which may have a prominent role in affective disorders).

As an exploratory study, we did not control for multiple comparisons, and (even limiting the number of regression models by initially conducting univariate analyses to focus on the potentially indicative proteins) several models were conducted, increasing the possibility of type 1 errors. The reporting of effect sizes in addition to *P*-values serves to aid interpretation of the effects observed. Future studies may consider these results to guide the investigation of candidate biomarkers of cognition in populations with bipolar disorder. Future work should also attempt to build on this work by assessing longitudinal relationships, as this cross-sectional study is not able to infer causality of association.

### Potential mechanisms for cognitive impairment in bipolar disorder

In addition to neurogenesis, associations between reduced hippocampal volume and cognitive impairment in bipolar disorder may be mediated by inflammation or neuronal toxicity,^66^ although longitudinal studies are needed to ascertain temporal associations between these putatively related phenomena. Oxidative stress may also implicate mitochondrial,^67^ HPA axis,^68^ monoamine^26^ and/or white matter^69,70^ dysfunctions, all of which have been linked with bipolar disorder and cognitive difficulties. Additionally, genetic interactions may provide additional support for some of the above relationships, e.g. IL-1^71^ or IL-6^72^ polymorphisms.

It is worth noting that both inflammatory proteins and cognitive impairments indicate dysregulations that persist into periods of recovery, but are exacerbated during acute affective episodes. However, the proteomic markers identified relate to cognitive and affective illness characteristics that manifest in individuals with bipolar disorder, and clearly inflammatory and neurotrophic systems play a role in the key features of bipolar disorder. Relevant interventional research has focused thus far on the antidepressant and antimanic effects of anti-inflammatory medications in individuals with mood disorders,^73^ but the same agents have also shown pro-cognitive and neurogenic effects.^74^ Despite the promise of anti-inflammatory treatments for affective episodes, the use of minocycline and celecoxib was not supported in the largest study of people with bipolar disorder in a depressive episode to date,^75^ although there was no apparent measurement of fundamental cognitive outcomes. In addition to translational research of pharmacological treatments that regulate inflammatory activity, it may likewise be informative to assess the effects of pharmacological and psychosocial pro-cognitive interventions on inflammatory and neurotrophic protein outcomes in populations with bipolar disorder.

In conclusion, this study has provided insight into possible biological markers of cognitive impairment in individuals with bipolar disorder currently free from affective symptoms. The proteins implicated include some established markers, such as VEGF-C and bFGF, and some novel putative targets, including IL-7 and PlGF. In addition to further investigation of putative neurobiological underpinnings of cognitive dysfunction in larger and longitudinal studies, future work should consider the implications of this work for potentially detecting and treating cognitive impairment in those with bipolar disorder, which is an emerging field that is gaining momentum.^8,25^ Anti-inflammatory treatments appear to have some antidepressant effects, and these may also be ameliorating cognitive impairments observed in bipolar depression (where cognitive difficulties are more pronounced than in euthymia).

This exciting area of investigation is still in its infancy, and as such, more clinical studies are needed to more fully understand the nature and mechanisms underlying the relationship of inflammation and growth factors with cognitive deficits in bipolar disorder. This could then guide future clinical trials involving existing or potentially novel anti-inflammatory interventions addressing cognition, rather than just mood, more directly.

## Data Availability

Data availability requests should be submitted to the corresponding author.

## Supplementary material

For supplementary material accompanying this paper visit http://dx.doi.org/10.1192/bjo.2021.966. click here to view supplementary material
