# Supplementary material for: Inflammatory biomarkers and cognitive functioning in individuals with euthymic bipolar disorder: exploratory study
Source: BJPsych Open. 2021 Jul 9;7(4):e126. doi: 10.1192/bjo.2021.966 (PMC8281256; doi:10.1192/bjo.2021.966)
Supplement: Supplementary file 1 [file S2056472421009662sup001.docx]

**Supplement 1: Descriptive protein data**

| **Protein** | **Mean** | **SD** | **Completeness** |
| --- | --- | --- | --- |
| BDNF | 1986.868 | 1552.494 | Complete |
| bFGF | 28.514 | 30.280 | Complete |
| CRP | 3.463 | 4.047 | Complete |
| Eotaxin ^a^ | 120.599 | 45.800 | Complete |
| Eotaxin3 | 20.466 | 8.687 | Complete |
| Flt1 | 76.704 | 13.932 | Complete |
| ICAM1 ^a^ | 0.596 | 0.210 | Complete |
| IFNy | 5.595 | 6.916 | 1 missing. Imputed LLOD/2 (0.025) |
| IL10 | 0.312 | 0.284 | 1 missing. Imputed LLOD/2 (0.005) |
| IL12 | 176.910 | 108.679 | Complete |
| IL15 | 3.028 | 0.450 | Complete |
| IL16 | 140.836 | 48.305 | Complete |
| IL17 | 2.658 | 1.450 | Complete |
| IL1a | 10.009 | 7.270 | Complete |
| IL6 | 1.019 | 1.565 | Complete |
| IL7 | 3.217 | 2.405 | Complete |
| IL8 | 3.781 | 3.653 | Complete |
| IP10 | 309.590 | 201.340 | Complete |
| MCP1 | 83.744 | 30.190 | Complete |
| MCP4 | 85.490 | 21.639 | Complete |
| Mip1a | 22.570 | 12.725 | Complete |
| Mip1b | 53.320 | 26.102 | Complete |
| Plgf | 5.551 | 1.769 | Complete |
| SAA ^a^ | 6.272 | 7.704 | Complete |
| TARC | 60.826 | 66.340 | 5 missing. Imputed LLOD/2 (0.085) |
| Tie2 | 7262.932 | 2061.002 | Complete |
| TNFa | 2.368 | 1.175 | Complete |
| TNFb | 0.355 | 0.347 | 2 missing. Imputed LLOD/2 (0.02) |
| VCAM1 ^a^ | 0.601 | 0.239 | Complete |
| VEGFC | 69.480 | 43.133 | Complete |
| VEGFD | 308.629 | 82.420 | Complete |
| VEGF | 93.967 | 69.276 | Complete |

Proteins with >20% undetected: GMCSF, IL-12p70, IL-13, IL-1b, IL-2, IL-4, IL-5, IFNa.

SD = standard deviation, LLOD = lower limit of detection.

^a^ pg/ml converted to mg/L. All other protein levels expressed as pg/ml

**Supplement 2: Protein comparisons with non-biological factors**

1. *Continuous comparisons (Spearman’s correlations)*

| **Biomarker** | | **Age** | **Meds.** | **BMI** | **HR-QOL** | **Episodes** | **HAMD** | **YMRS** | **FAST** | **HAMA** | **CTQ** |
| --- | --- | --- | --- | --- | --- | --- | --- | --- | --- | --- | --- |
| BDNF | r  p | -0.087  0.576 | -0.350  0.020 ** | 0.210  0.181 | -0.078  0.615 | -0.142  0.358 | -0.239  0.119 | 0.021  0.894 | -0.197  0.199 | 0.003  0.986 | -0.158  0.307 |
| bFGF | r  p | 0.009  0.952 | -0.064  0.678 | 0.212  0.178 | 0.110  0.475 | -0.059  0.704 | -0.186  0.226 | -0.166  0.282 | -0.104  0.503 | 0.174  0.259 | -0.058  0.709 |
| IL-16 | r  p | 0.068  0.660 | 0.110  0.478 | -0.017  0.914 | 0.403  0.007 *** | -0.121  0.436 | 0.062  0.692 | 0.085  0.582 | 0.061  0.692 | 0.154  0.318 | 0.262  0.086* |
| IL-6 | r  p | 0.0217  0.158 | 0.106  0.491 | 0.178  0.259 | 0.445  0.002 *** | 0.053  0.734 | 0.228  0.137 | 0.229  0.135 | 0.494  0.001**** | 0.179  0.244 | 0.218  0.154 |
| IL-7 | r  p | -0.019  0.905 | -0.259  0.090 * | 0.164  0.300 | -0.022  0.887 | -0.163  0.290 | -0.209  0.174 | -0.079  0.612 | -0.135  0.383 | 0.060  0.697 | -0.215  0.162 |
| Mip1b | r  p | 0.160  0.298 | 0.075  0.627 | 0.241  0.124 | 0.125  0.418 | 0.035  0.823 | -0.059  0.703 | -0.011  0.946 | 0.183  0.235 | -0.178  0.249 | 0.091  0.559 |
| PlGF | r  p | 0.604  <.001**** | 0.332  0.028** | -0.042  0.793 | 0.261  0.087* | 0.155  0.316 | 0.152  0.325 | 0.009  0.956 | 0.153  0.321 | 0.115  0.457 | -0.073  0.637 |
| TNFb | r  p | -0.171  0.267 | 0.057  0.712 | -0.252  0.108 | -0.068  0.661 | -0.324  0.032** | 0.060  0.700 | -0.132  0.392 | 0.152  0.325 | 0.095  0.538 | -0.034  0.828 |
| VEGFC | r  p | 0.093  0.548 | 0.109  0.482 | 0.05  0.755 | 0.065  0.676 | -0.058  0.708 | 0.062  0.688 | -0.110  0.476 | 0.201  0.192 | 0.217  0.158 | -0.112  0.469 |

1. *Dichotomous comparisons (independent samples t test)*

| **Biomarker** | | **Gender**  *(female / male)* | **Bipolar type**  *(type II / type I)* | **Physical illness**  *(yes / no)* | **Smoking**  *(no / yes)* | **Alcohol**  *(higher / lower)* |
| --- | --- | --- | --- | --- | --- | --- |
| *BDNF* | t^a^  p [95%CI] | 0.035  0.973 [-0.243, 0.252] | -1.677  0.101 [-0.298, 0.028] | -1.383  0.174 [-0.277, 0.052] | 0.454  0.652 [-0.143, 0.227] | -1.025  0.311 [-0.250, 0.082] |
| *bFGF* | t  p [95%CI] | -0.764  0.449 [-0.395, 0.178] | -1.035  0.307 [-0.375, 0.121] | -0.487  0.629 [-0.332, 0.202] | 0.148  0.883 [-0.274, 0.317] | 0.609  0.546 [-0.186, 0.347] |
| *IL-16* | t  p [95%CI] | -0.525  0.603 [-0.117, 0.069] | -2.158 **  0.037 [-0.169, -0.006] | 0.663  0.511 [-0.058, 0.114] | -0.004  0.997 [-0.095, 0.095] | -0.390  0.699 [-0.103, 0.069] |
| *IL-6* | t^a^  p [95%CI] | 0.715  0.479 [-0.160, 0.335] | -1.568  0.124 [-0.369, 0.046] | 1.716 *  0.094 [-0.031, 0.375] | -2.509 **  0.016 [-0.534, -0.058] | -0.107  0.915 [-0.243, 0.219] |
| *IL-7* | t^a^  p [95%CI] | 0.186  0.853 [-0.189, 0.228] | -1.416  0.164 [-0.322, 0.056] | -1.444  0.156 [-0.324, 0.054] | 1.069  0.291 [-0.099, 0.322] | -0.612  0.545 [-0.027, 0.146] |
| *Mip1b* | t^a^  p [95%CI] | -3.205 ***  0.003 [-0.274, -0.062] | -0.641  0.525 [-0.144, 0.074] | -1.117  0.270 [-0.168, 0.048] | 1.285  0.206 [-0.043, 0.194] | 1.625  0.112 [-0.021, 0.192] |
| *PlGF* | t^a^  p [95%CI] | -2.556 **  0.014 [-0.178, -0.021] | -1.003  0.322 [-0.116, 0.039] | -0.783  0.438 [-0.108, 0.047] | 2.942 ***  0.006 [0.028, 0.153] | 1.352  0.189 [-0.029, 0.141] |
| *TNFb* | t^a^  p [95%CI] | 0.176  0.861 [-0.237, 0.282] | -0.599  0.552 [-0.311, 0.169] | -0.791  0.433 [-0.333, 0.145] | 0.001  0.999 [-0.266, 0.266] | 0.206  0.837 [-0.216, 0.265] |
| *VEGFC* | t^a^  p [95%CI] | -0.306  0.761 [-0.172, 0.127] | -0.629  0.533 [-0.167, 0.088] | -0.165  0.870 [-0.150, 0.128] | -0.476  0.637 [-0.189, 0.117] | -0.286  0.776 [-0.159, 0.119] |

* = p<0.1, ** = p<0.05, *** = p<0.01, **** = p<0.001

BDNF = brain derived neurotrophic factor, bFGF = basic fibroblast growth factor, IL = interleukin, Mip1b = macrophage inflammatory protein 1b, PlGF = placental growth factor, TNFb = tumour necrosis factor beta, VEGFC = vascular endothelial growth factor C. Meds = number of medications currently taken, BMI = body mass index, HR-QOL = health-related quality of life (EQ5D score), episodes = number of lifetime affective episodes, HAMD = subsyndromal depressive symptom severity, YMRS = subsyndromal manic symptom severity, FAST = functional impairment, HAMA = anxiety severity, CTQ = childhood trauma severity.

A: directionality of association is denoted by positive/negative correlation and direction of abbreviations explained above.

B: directionality of association is denoted via the header (positive/negative) whereby a positive t value indicates higher in women, bipolar type II, physically ill participants, non-smokers, higher alcohol intake and a negative t value denotes the reverse direction of association.

**Supplement 3: Inter-correlations between proteins**

| **Biomarker** |  | BDNF | bFGF | IL-16 | IL-6 | IL-7 | Mip1b | PlGF | TNFb | VEGFC |
| --- | --- | --- | --- | --- | --- | --- | --- | --- | --- | --- |
| BDNF | *r* |  |  |  |  |  |  |  |  |  |
|  | *p* |  |  |  |  |  |  |  |  |  |
| bFGF | *r* | 0.548 |  |  |  |  |  |  |  |  |
|  | *p* | <0.001 *** |  |  |  |  |  |  |  |  |
| IL-16 | *r* | 0.212 | 0.362 |  |  |  |  |  |  |  |
|  | *p* | 0.168 | 0.016* |  |  |  |  |  |  |  |
| IL-6 | *r* | 0.008 | 0.131 | 0.223 |  |  |  |  |  |  |
|  | *p* | 0.96 | 0.395 | 0.146 |  |  |  |  |  |  |
| IL-7 | *r* | 0.789 | 0.660 | 0.288 | -0.110 |  |  |  |  |  |
|  | *p* | <0.001 *** | <0.001 *** | 0.058 | 0.479 |  |  |  |  |  |
| Mip1b | *r* | 0.137 | 0.062 | 0.054 | 0.096 | 0.155 |  |  |  |  |
|  | *p* | 0.374 | 0.690 | 0.729 | 0.536 | 0.314 |  |  |  |  |
| PlGF | *r* | -0.034 | 0.300 | 0.490 | 0.108 | 0.225 | 0.320 |  |  |  |
|  | *p* | 0.827 | 0.048 * | 0.001 *** | 0.484 | 0.141 | 0.034 * |  |  |  |
| TNFb | *r* | -0.004 | 0.097 | 0.371 | 0.001 | 0.248 | 0.058 | 0.170 |  |  |
|  | *p* | 0.981 | 0.532 | 0.013 * | 0.996 | 0.105 | 0.709 | 0.269 |  |  |
| VEGFC | *r* | 0.481 | 0.589 | 0.395 | 0.092 | 0.687 | 0.142 | 0.378 | 0.349 |  |
|  | *p* | 0.001*** | <0.001 *** | 0.008 ** | 0.554 | <0.001 *** | 0.357 | 0.011 * | 0.02 * |  |

* = p<0.05, ** = 0<0.01, *** = p<0.001

BDNF = brain derived neurotrophic factor, bFGF = basic fibroblast growth factor, IL = interleukin, Mip1b = macrophage inflammatory protein 1b, PlGF = placental growth factor, TNFb = tumour necrosis factor beta, VEGFC = vascular endothelial growth factor C.

**Supplement 4: Post-hoc sensitivity analysis after removal of two participants with inflammatory conditions / medications**

The results that were affected after removing these two participants comprised:

1. IL-6 was no longer potentially associated with impairment group (p = 0.133)
2. TNFb was no longer potentially associated with impairment group (p = 0.141)
3. bFGF was no longer potentially associated with impairment group (p = 0.156) although remained associated with continuous cognitive performance.
4. IL-16 was no longer potentially associated with impairment group (p = 0.175) although remained associated with continuous cognitive performance.
5. Two additional cytokines were now associated with bipolar subtype at p < 0.1, higher in those with BD type I: BDNF t(40) = 1.938, p = 0.055; IL-7 t(40) = 1.755, p = 0.084
6. IL-6 was no longer associated with physical illness (p = 0.181).
7. PlGF was no longer associated with health-related quality of life (p = 0.187).

**Table A: Multivariable logistic regressions predicting cognitive impairment group (akin to table 3)**

| **Biomarker** | **Model r^2^** | **Model X^2^** | **Model p** | **IVs** | **IV OR** | **95% CI** | **p** | **Notes** |
| --- | --- | --- | --- | --- | --- | --- | --- | --- |
| BDNF | 0.305 | 10.865 | **0.012** | BDNF  N medications  **Smoking** | 0.224  1.287  7.939 | 0.013, 3.739  0.908, 1.823  1.459, 43.20 | 0.246  0.122  **0.002** | Almost identical results to n=44; table 3 and results unaffected by adding bipolar subtype (the latter non-significant). |
| bFGF | 0.232 | 8.002 | 0.018 | bFGF  **Smoking** | 0.328  6.063 | 0.062, 1.729  1.302, 28.23 | 0.202  **0.013** | Almost identical results to n=44; table 3 |
| IL-16^a^ | 0.276 | 9.712 | 0.084 | IL-16  HRQOL  CTQ  Bipolar type  **Smoking** | 0.096  0.701  1.011  1.063  8.719 | 0.000, 46.04  0.385, 1.277  0.965, 1.060  0.226, 4.994  1.493, 50.92 | 0.575  0.357  0.673  0.948  **0.011** | Almost identical results to n=44; table 3 |
| IL-6 | 0.400 | 14.904 | **0.011** | IL-6  **HRQOL**  **Smoking**  Physical illness  **FAST** | 2.587  0.427  8.751  0.300  1.096 | 0.199, 33.58  0.217, 0.839  1.128, 67.88  0.054, 1.677  0.979, 1.226 | 0.412  **0.002**  **0.033**  0.160  **0.078** | NB IL-6 no longer associated with cognition in univariate tests. Model almost identical results to n=44 (table 3) though smoking was not previously significant; FAST was previously significant). Removing physical illness as a covariate did not much affect findings although FAST was no longer associated. |
| IL-7 | 0.446 | 17.029 | **<0.001** | **IL-7**  N medications  **Smoking** | 0.025  1.256  7.856 | 0.001, 0.518  0.869, 1.816  1.214, 50.83 | **0.009**  0.286  **0.004** | Almost identical results to n=44; table 3 and results unaffected by adding bipolar subtype (the latter non-significant). |
| Mip1b | 0.268 | 9.391 | 0.025 | Mip1b  Gender  **Smoking** | 0.021  1.066  5.297 | 0.000, 2.396  0.203, 5.598  1.095, 25.63 | 0.101  0.919  **0.016** | Overall model slightly more explanatory than n=44 (table 3); individual IVs almost identical. |
| PlGF ^a^ | 0.544 | 21.940 | **0.001** | **PlGF**  **N medications**  HRQOL  Gender  **Smoking**  Age | 0.000  1.801  0.581  1.513  21.04  1.073 | 0.000, 0.313  1.089, 2.980  0.309, 1.090  0.177, 12.95  1.815, 243.8  0.972, 1.185 | **0.018**  **0.010**  0.070  0.626  **0.005**  0.202 | Overall model slightly more explanatory than n=44 (table 3); individual IVs similar without change in significance thresholds. Removing HRQOL as a covariate reduced the overall model strength (r2 = 0.479) with PlGF, number of medications and smoking remaining independently associated. |
| TNFb | 0.262 | 9.156 | **0.027** | **TNFb**  N episodes  **Smoking** | 0.220  0.989  6.869 | 0.033, 1.449  0.951, 1.029  1.391, 33.93 | **0.057**  0.558  **0.011** | NB TNFb no longer associated with cognition in univariate tests. Model almost identical results to n=44 (table 3) although TNFb no longer statistically significant (was p = 0.03). |
| VEGFC | 0.320 | 11.474 | **0.003** | **VEGFC**  **Smoking** | 0.026  8.967 | 0.001, 0.717  1.535, 52.40 | **0.008**  **0.004** | Almost identical results to n=44; table 3 |

Multivariable logistic regressions did not indicate a significant concern of collinearity within any of the models (Hosmer-Lemeshow test). NB the p values provided are following bootstrapping. Bold text indicates significance at p < 0.05.

IV = independent variables, OR = odds ratio, CI = confidence interval, BDNF = brain derived neurotrophic factor, bFGF = basic fibroblast growth factor, IL = interleukin, Mip1b = macrophage inflammatory protein 1b, PlGF = placental growth factor, TNFb = tumour necrosis factor beta, VEGFC = vascular endothelial growth factor C, n = number, HR-QOL = health-related quality of life (EQ5D score), FAST = functional impairment, CTQ = childhood trauma severity.

^a^ For underpowered models (those containing > 1 IV per 10 participants i.e. >4 IV’s in total), regressions were re-run only containing covariates that were significant at p < 0.05 (between inflammatory and non-biological, or cognitive and non-biological) i.e. IL-16 (removing CTQ) and PlGF (removing number of medications), although the latter overall model was weakened by this removal.

**Table B: Multivariable linear regressions predicting global cognitive performance (akin to table 4)**

| **Biomarker** | **Model adjusted r^2^** | **Model F** | **Model p** | **IVs** | **IV s*Beta*** | **95% CI** | **p** | **Notes** |
| --- | --- | --- | --- | --- | --- | --- | --- | --- |
| BDNF | 0.015 | 1.207 | 0.320 | BDNF  N medications  FAST | 0.089  -0.122  -0.189 | -0.529, 0.788  -0.113, 0.048  -0.035, 0.007 | 0.513  0.431  0.210 | Almost identical results to n=44; table 4 and results unaffected by adding bipolar subtype (the latter non-significant). |
| bFGF | 0.124 | 3.913 | **0.028** | **bFGF**  **FAST** | 0.325  -0.246 | 0.091, 0.834  -0.034, 0.001 | **0.017**  **0.072** | Similar results to n=44 (table 4) but FAST no longer significant at p < 0.05 (p = 0.037). |
| IL-16^a^ | 0.080 | 1.712 | 0.157 | IL-16  HRQOL  CTQ  Bipolar type  **FAST** | 0.249  0.234  -0.118  -0.031  -0.373 | -0.573, 2.902  -0.111, 0.318  -0.017, 0.014  -0.462, 0.388  -0.044, -0.004 | 0.248  0.447  0.593  0.861  **0.030** | Almost identical results to n=44; table 4 |
| IL-6 | 0.062 | 1.538 | 0.203 | IL-6  HRQOL  Smoking  Physical illness  **FAST** | -0.056  0.418  0.105  -0.177  -0.383 | -0.616, 0.669  -0.043, 0.335  -0.358, 0.703  -0.530, 0.181  -0.049, -0.003 | 0.749  0.099  0.605  0.256  **0.028** | NB IL-6 no longer associated with cognition in univariate tests. Model almost identical results to n=44 (table 4). Removing physical illness as a covariate did alter findings. |
| IL-7 | 0.154 | 3.482 | **0.025** | **IL-7**  N medications  FAST | 0.377  -0.064  -0.202 | 0.290, 1.297  -0.098, 0.070  -0.033, 0.006 | **0.007**  0.664  0.146 | Almost identical results to n=44 (table 4). Results largely unaffected by adding bipolar subtype (although model strength reduced to p=0.055; BD subtype non-significant). |
| Mip1b | 0.006 | 1.085 | 0.367 | Mip1b  Gender  FAST | 0.115  -0.035  -0.269 | -0.884, 1.522  -0.515, 0.333  -0.038, 0.006 | 0.493  0.814  0.086 | Model similar to n=44 (table 4) although FAST no longer significant at p < 0.05 (was p = 0.028). |
| PlGF | 0.186 | 2.335 | **0.046** | **PlGF**  N medications  HRQOL  Gender  Smoking  Age  FAST | 0.438  -0.199  0.329  0.021  0.141  -0.318  -0.321 | 0.724, 4.696  -0.151, 0.041  -0.018, 0.252  -0.396, 0.484  -0.313, 0.642  -0.036, 0.006  -0.043, 0.002 | **0.036**  0.250  0.085  0.895  0.449  0.182  0.064 | Model similar to n=44 (table 4) although FAST no longer significant at p < 0.05 (was p = 0.039). Removing HRQOL as a covariate reduced the overall model strength (adj r2 = 0.121, p = 0.101) with PlGF reduced to a trend (p = 0.051). |
| TNFb | 0.031 | 1.433 | 0.248 | TNFb  N episodes  FAST | 0.207  0.018  -0.292 | -0.381, 0.775  -0.011, 0.017  -0.044, 0.000 | 0.233  0.910  0.068 | NB TNFb no longer associated with cognition in univariate tests. Model almost identical results to n=44 (table 4) but FAST no longer significant at p < 0.05 (was p = 0.031). |
| VEGFC | 0.168 | 5.145 | **0.010** | **VEGFC**  **FAST** | 0.392  -0.329 | 0.388, 1.899  -0.042, -0.004 | **0.009**  **0.021** | Almost identical results to n=44 (table 4). |

Multivariable linear regressions did not indicate a significant concern of collinearity within any of the models (Durbin-Watson value between 1 and 3.) Bold text indicates significance at p < 0.05.

IV = independent variables, sBeta = standardized beta value, CI = confidence interval, BDNF = brain derived neurotrophic factor, bFGF = basic fibroblast growth factor, IL = interleukin, Mip1b = macrophage inflammatory protein 1b, PlGF = placental growth factor, TNFb = tumour necrosis factor beta, VEGFC = vascular endothelial growth factor C, n = number, HR-QOL = health-related quality of life (EQ5D score), FAST = functional impairment, CTQ = childhood trauma severity.

^a^ For underpowered models (those containing > 1 IV per 10 participants i.e. >4 IV’s in total), regressions were re-run only containing covariates that were significant at p < 0.05 (between inflammatory and non-biological, or cognitive and non-biological) i.e. IL-16 (removing CTQ) and PlGF (removing number of medications), with these not significantly affecting results.

**Supplement 5: Inflammatory associations with individual cognitive tests**

|  | | **Processing speed** | | **Working memory** | **Verbal learning** | **Verbal memory** | **Executive functioning** | | **Verbal fluency** |
| --- | --- | --- | --- | --- | --- | --- | --- | --- | --- |
|  |  | *Digit symbol substitution test^1^* | *Symbol search^1^* | *Digit span^1^* | *Verbal paired associates I^2^* | *Verbal paired associates II^2^* | *The hotel test^3^* | *Matrix Reasoning^4^* | *FAS test^5^* |
| BDNF | r | 0.077 | 0.289 | -0.033 | 0.207 | 0.297 | 0.195 | 0.134 | -0.213 |
|  | p | 0.617 | 0.057 | 0.832 | 0.177 | 0.050* | 0.205 | 0.387 | 0.165 |
| bFGF | r | 0.219 | 0.317 | 0.270 | 0.300 | 0.340 | 0.131 | 0.266 | -0.020 |
|  | p | 0.153 | 0.036^*^ | 0.076 | 0.048^*^ | 0.024^*^ | 0.396 | 0.081 | 0.897 |
| IL-16 | r | 0.221 | 0.301 | -0.130 | 0.302 | 0.326 | 0.229 | 0.192 | -0.016 |
|  | p | 0.150 | 0.047^*^ | 0.401 | 0.046^*^ | 0.031^*^ | 0.135 | 0.213 | 0.917 |
| IL-6 | r | -0.185 | -0.095 | -0.164 | 0.059 | 0.004 | -0.274 | -0.215 | -0.144 |
|  | p | 0.230 | 0.540 | 0.288 | 0.704 | 0.980 | 0.072 | 0.161 | 0.352 |
| IL-7 | r | 0.321 | 0.405 | 0.257 | 0.339 | 0.429 | 0.238 | 0.286 | 0.015 |
|  | p | 0.033^*^ | 0.006^**^ | 0.093 | 0.025^*^ | 0.004^**^ | 0.120 | 0.060 | 0.922 |
| Mip1b | r | 0.049 | 0.043 | 0.172 | 0.030 | -0.037 | 0.067 | -0.026 | -0.005 |
|  | p | 0.753 | 0.779 | 0.263 | 0.845 | 0.810 | 0.667 | 0.865 | 0.977 |
| PlGF | r | 0.198 | 0.171 | 0.096 | 0.055 | 0.037 | 0.178 | 0.088 | 0.193 |
|  | p | 0.198 | 0.268 | 0.537 | 0.722 | 0.814 | 0.247 | 0.571 | 0.210 |
| TNFb | r | 0.223 | 0.223 | 0.006 | 0.051 | -0.007 | 0.106 | 0.012 | 0.023 |
|  | p | 0.146 | 0.145 | 0.970 | 0.744 | 0.967 | 0.492 | 0.936 | 0.883 |
| VEGFC | r | 0.256 | 0.416 | 0.310 | 0.230 | 0.258 | 0.070 | 0.232 | 0.100 |
|  | p | 0.094 | 0.005^**^ | 0.041^*^ | 0.133 | 0.091 | 0.653 | 0.130 | 0.517 |

* = p<0.05, ** = 0<0.01

*^1^* Wechsler Adult Intelligence Scale (WAIS), *^2^* Wechsler Memory Scale (WMS), *^3^* Hotel Test of multitasking and planning, *^4^* Wechsler Abbreviated Scale of Intelligence (WASI), *^4^* Delis-Kaplan executive function system.

BDNF = brain derived neurotrophic factor, bFGF = basic fibroblast growth factor, IL = interleukin, Mip1b = macrophage inflammatory protein 1b, PlGF = placental growth factor, TNFb = tumour necrosis factor beta, VEGFC = vascular endothelial growth factor C.
